# Enhancing Performance of SSVEP-Based Visual Acuity via Spatial Filtering

**DOI:** 10.3389/fnins.2021.716051

**Published:** 2021-08-19

**Authors:** Xiaowei Zheng, Guanghua Xu, Chengcheng Han, Peiyuan Tian, Kai Zhang, Renghao Liang, Yaguang Jia, Wenqiang Yan, Chenghang Du, Sicong Zhang

**Affiliations:** ^1^School of Mechanical Engineering, Xi’an Jiaotong University, Xi’an, China; ^2^State Key Laboratory for Manufacturing Systems Engineering, Xi’an Jiaotong University, Xi’an, China

**Keywords:** visual acuity, steady-state visual evoked potential, spatial filtering, multielectrode signals combination, canonical correlation analysis

## Abstract

The purpose of this study was to enhance the performance of steady-state visual evoked potential (SSVEP)-based visual acuity assessment with spatial filtering methods. Using the vertical sinusoidal gratings at six spatial frequency steps as the visual stimuli for 11 subjects, SSVEPs were recorded from six occipital electrodes (O1, Oz, O2, PO3, POz, and PO4). Ten commonly used training-free spatial filtering methods, i.e., native combination (single-electrode), bipolar combination, Laplacian combination, average combination, common average reference (CAR), minimum energy combination (MEC), maximum contrast combination (MCC), canonical correlation analysis (CCA), multivariate synchronization index (MSI), and partial least squares (PLS), were compared for multielectrode signals combination in SSVEP visual acuity assessment by statistical analyses, e.g., Bland–Altman analysis and repeated-measures ANOVA. The SSVEP signal characteristics corresponding to each spatial filtering method were compared, determining the chosen spatial filtering methods of CCA and MSI with a higher performance than the native combination for further signal processing. After the visual acuity threshold estimation criterion, the agreement between the subjective Freiburg Visual Acuity and Contrast Test (FrACT) and SSVEP visual acuity for the native combination (0.253 logMAR), CCA (0.202 logMAR), and MSI (0.208 logMAR) was all good, and the difference between FrACT and SSVEP visual acuity was also all acceptable for the native combination (−0.095 logMAR), CCA (0.039 logMAR), and MSI (−0.080 logMAR), where CCA-based SSVEP visual acuity had the best performance and the native combination had the worst. The study proved that the performance of SSVEP-based visual acuity can be enhanced by spatial filtering methods of CCA and MSI and also recommended CCA as the spatial filtering method for multielectrode signals combination in SSVEP visual acuity assessment.

## Introduction

Visual acuity, one of the most necessary parameters to test visual function, is a measure of the spatial resolution of the visual processing. In general, it is mainly tested by psychophysical methods, e.g., Sloan letters and tumbling E charts (Ricci et al., 1998). However, these methods require the subjects to have sufficient intelligence to comply with the test process and are hard for preverbal or infantile children, the mentally disabled, and malingerers (Incesu and Sobaci, 2011; Zheng et al., 2020c).

Noninvasive electroencephalography (EEG), e.g., steady-state visual evoked potentials (SSVEPs), has been proved to provide an alternative method to estimate visual acuity objectively (Regan, 1973; Norcia et al., 2015). By varying the spatial frequency of the visual stimuli, visual acuity can be measured by a threshold determination criterion by establishing the mathematical model between spatial frequency and SSVEP signals (Hamilton et al., 2021a). Besides, previous studies proved that a larger number of posterior electrodes was relevant to optimize visual function assessment (Hemptinne et al., 2018) and recommended multielectrode montage, e.g., six-electrode of O1, Oz, O2, PO3, POz, and PO4 (Zheng et al., 2020a, 2021), rather than single-electrode in SSVEP visual acuity assessment (Hamilton et al., 2021b). However, in SSVEP visual acuity assessment, SSVEPs are mainly collected at only one active electrode, e.g., Oz at the midline over the occiput (McBain et al., 2007; Odom et al., 2016; Ridder, 2019), except for some other electrode montages, e.g., the bipolar electrodes of Oz and O1 (Norcia and Tyler, 1985a, b; Skoczenski and Norcia, 1999), which was sometimes used to enhanced signal-to-noise-ratio (SNR), especially close to the threshold (Hamilton et al., 2021b).

The spatial filtering technique combining the multielectrode signals into single- or multichannel signals offers a better method for extracting SSVEP features and eliminating nuisance signals in SSVEP studies (Yan et al., 2018). Since scalp EEG is usually regarded to be a linear mixture of multiple time series from various cortical sources (Onton et al., 2006), the weight coefficients can be applied for multielectrode scalp EEG signals to estimate the cortical source activities (Nakanishi et al., 2018b). On the basis of this idea, several methods of extracting optimal spatial filters to reconstruct source activities from scalp EEG signals have been carried out to enhance the SNR of SSVEPs. For instance, the basic spatial filtering methods [e.g., Laplacian combination (Friman et al., 2007) and common average reference (CAR) (Zheng et al., 2020d)] and the model-based spatial filtering methods [e.g., minimum energy combination (MEC) (Friman et al., 2007), canonical correlation analysis (CCA) (Bin et al., 2009; Zheng et al., 2020b; Li et al., 2021), and multivariate synchronization index (MSI) (Zhang et al., 2014a)] have been applied to improve the performance of SSVEPs. However, to date, little is known about whether there is an enhancement of the spatial filtering technique from multielectrode signals on SSVEP visual acuity.

On the basis, in this study, 10 commonly used training-free spatial filtering methods, i.e., native combination (i.e., single-electrode) (Friman et al., 2007), bipolar combination (Hamilton et al., 2021b), Laplacian combination, average combination (Friman et al., 2007), CAR, MEC, maximum contrast combination (MCC) (Friman et al., 2007), CCA, MSI, and partial least squares (PLS) (Ge et al., 2017), were compared for multielectrode signals combination in SSVEP visual acuity assessment. First, SSVEPs were induced by the vertical sinusoidal gratings at six spatial frequency steps and recorded from six occipital electrodes (O1, Oz, O2, PO3, POz, and PO4) for 11 subjects. Next, the SSVEP signal characteristics corresponding to each spatial filtering method were compared to determine the chosen spatial filtering methods with good performance for further signal processing. Then, SSVEP visual acuity can be obtained by the threshold estimation criterion for each chosen spatial filtering method, and the statistical analyses, e.g., Bland–Altman analysis and repeated-measures ANOVA, were used to explore the performance of the spatial filtering technique from multielectrode signals on SSVEP visual acuity. The main purpose of this study was to enhance the performance of SSVEP visual acuity with spatial filtering methods.

## Materials and Methods

### SSVEP Model

For the visual stimulus with a temporal frequency of *f*, the SSVEP signal, *y*_*i*_(*t*), measured as the voltage between a reference electrode and the *i*th electrode at time *t*, can be modeled as (Friman et al., 2007; Zerafa et al., 2018):

(1) $$y_{i}\left(t\right)=\sum_{h=1}^{N_{h}}a_{i,h}\sin\left(2\pi hft+\phi_{i,h}\right)+e_{i}\left(t\right)$$

This linear model consists of two parts: the evoked SSVEP response signal and the noise signal. The evoked SSVEP response consists of many sinusoids with the frequency given by the stimulus frequency *f* and its harmonic frequencies. *N*_*h*_ is the number of harmonic frequencies. Each sinusoid is determined by its specific amplitude *a*_*i,h*_ and phase ϕ_*i,h*_. The noise signal *e*_*i*_(*t*) is composed of other signals that are unrelated to SSVEP response, such as electromyography (EMG), electrooculogram (EOG), and other components.

Hence, the SSVEP signal for a time segment of *N*_*t*_ samples with a sampling frequency *F*_*s*_ can be defined in vector form:

(2) $${\text{y}}_{i}={\text{X}}_{f}{\text{g}}_{i}+{\text{e}}_{i}$$

where ***y_i_*** = [*y*_*i*_(1),…,*y*_*i*_(*N*_*t*_)]^T^ ∈ *ℝ*^*N*_*t*_×1^ contains the SSVEP signal of the *i*th electrode in one segment of *N*_*t*_ samples, and ***e_i_*** ∈ *ℝ*^*N*_*t*_×1^ is the noise vector. The SSVEP reference signals model ***X_f_*** ∈ *ℝ*^*N*_*t*_×2*N*_*h*_^ is defined by Nakanishi et al. (2018b):

(3) $${\text{X}}_{f}={\left(\begin{array}{c} \text{sin}\left(2\pi f\frac{m}{F_{s}}\right)\\ \text{cos}\left(2\pi f\frac{m}{F_{s}}\right)\\ \vdots \\ \text{sin}\left(2\pi N_{h}f\frac{m}{F_{s}}\right)\\ \text{cos}\left(2\pi N_{h}f\frac{m}{F_{s}}\right) \end{array}\right)}^{\text{T}},m=1,\ldots ,N_{t}.$$

The vector ***g_i_*** ∈ *ℝ*^2*N*_*h*_×1^ contains the corresponding amplitude *a*_*i,h*_ and phase ϕ_*i,h*_.

Finally, for SSVEP signals recorded from *N*_*e*_ electrodes, the model ***Y*** can be further defined as:

(4) $$\text{Y}={\text{X}}_{f}\text{G}+\text{E}$$

where ***Y*** = [***y***_1_,…,***y***_*N*_*e*__] ∈ *ℝ*^*N*_*t*_×*N*_*e*_^ contains the sampled SSVEP signals from all electrodes, with each column corresponding to an electrode. ***E*** ∈ *ℝ*^*N*_*t*_×*N*_*e*_^ is the noise matrix, and ***G*** ∈ *ℝ*^2*N*_*h*_×*N*_*e*_^ contains the amplitudes and phases for all sinusoids.

### Spatial Filtering Model

In SSVEPs, the method of linearly combining the multielectrode signals into single- or multichannel signals is called spatial filtering (Yan et al., 2018) to enhance the SNR of SSVEP response. Given *N*_*e*_-electrode SSVEP signals ***Y*** as expressed in **Equation (4)**, single-channel ***s*** ∈ *ℝ*^*N*_*t*_×1^ can be created by combining ***Y*** linearly using weights ***w*** ∈ *ℝ*^*N*_*e*_×1^ (Friman et al., 2007):

(5) s=Yw.

More generally, multichannel signals ***S*** can be created by combining ***Y*** linearly using weights ***W*** (Friman et al., 2007):

(6) S=YW

where ***S*** = [***s_1_***, ***s***_***N_c_***_] ∈ *ℝ*^*N*_*t*_×*N*_*c*_^ are the spatially filtered signals, and *N*_*c*_ is the number of the channels considered for further signal analysis. When *N*_*c*_ is 1, **Equation (5)** is the same as **Equation (6)**. ***W*** = [***w_1_***, …, ***w_N_c__***] ∈ *ℝ*^*N*_*e*_×*N*_*c*_^ is the weight matrix for spatial filtering. Below, 10 commonly used spatial filtering methods for choices of ***W*** were introduced.

### Spatial Filtering Methods

Here, we aimed to compare the effect on visual acuity assessment by SSVEPs with different spatial filtering methods to combine multielectrode signals into a single-channel signal. The visual acuity results depend on the SSVEP amplitude changes versus spatial frequencies (Zheng et al., 2020c), and the SSVEP amplitude is usually obtained from single-channel SSVEP by using Fourier analysis to transform an SSVEP signal from the time domain to the frequency domain and extracting the specific SSVEP amplitude at the fundamental frequency of the visual stimulus from the resulting spectrum (Hamilton et al., 2021a, b). Hence, here, we only focused on the single-channel spatial filtering methods, i.e., *N*_*c*_ = 1, and ***W*** = ***w*** ∈ *R*^*N*_*e*_×1^.

#### Native Combination

The native combination is also called the monopolar combination where only the SSVEP signals from one of the electrodes are analyzed (Friman et al., 2007; Zerafa et al., 2018). In the SSVEP analysis, the most used electrode is Oz (Yan et al., 2021; Zheng et al., 2020c). Assuming that the SSVEP signals from the Oz electrode are corresponding to the first column in *N*_*e*_-electrode SSVEP signals ***Y*** (same below), the spatial filtering weights ***w*** can be expressed as:

(7) $$\text{w}={\left[1,0,\ldots ,0\right]}^{\text{T}}.$$

#### Bipolar Combination

The bipolar combination is used to reduce the common noise signals by measuring the voltage of two closely placed electrodes (Friman et al., 2007). In SSVEP visual acuity assessment, the bipolar combination sometimes is also used (Hamilton et al., 2021b). According to the previous studies (Norcia and Tyler, 1985a, b), we chose the commonly used electrode pair (Oz–O1). Hence, assuming that the SSVEP signals from the O1 electrode are from the second column in ***Y***, ***w*** can be expressed as:

(8) $$\text{w}={\left[1,-1,0,\ldots ,0\right]}^{\text{T}}.$$

#### Laplacian Combination

The Laplacian combination is the improvement of the bipolar combination by using the mean voltage of the surrounding electrodes from one center electrode as the reference voltage (Hamilton et al., 2021b). Laplacian combination is mainly divided into two types in SSVEP visual acuity studies: one- and two-dimensional Laplacian combination (Hamilton et al., 2021b). One-dimensional Laplacian combination in SSVEP acuity studies is carried out by using voltage from Oz − 1/2(O1 + O2) as the signal (Bach and Heinrich, 2019; Knotzele and Heinrich, 2019; Kurtenbach et al., 2013). A two-dimensional Laplacian combination, i.e., the fourth Laplacian combination of Oz − 1/4(O1 + O2 + POz + Iz) (Hamilton et al., 2013), is also used in the relevant study. Here, assuming that the SSVEP signals from the O1, O2, POz, and Iz electrode are the second, the third, the fourth, and the fifth column in ***Y***, respectively, ***w*** for one-dimensional Laplacian combination can be expressed as:

(9) $$\text{w}={\left[1,-\frac{1}{2},-\frac{1}{2},0,\ldots ,0\right]}^{\text{T}}.$$

and ***w*** for two-dimensional Laplacian combination can be expressed as:

(10) $$\text{w}={\left[1,-\frac{1}{4},-\frac{1}{4},-\frac{1}{4},-\frac{1}{4},0,\ldots ,0\right]}^{\text{T}}.$$

#### Average Combination

The average combination is used by taking the average signals from all electrodes to amplify the SSVEP component and cancel the electrode-specific noise (Friman et al., 2007), where the weights ***w*** can be expressed as:

(11) $$\text{w}={\left[\frac{1}{N_{e}},\ldots ,\frac{1}{N_{e}}\right]}^{\text{T}}.$$

#### Common Average Reference

Common average reference, a commonly used spatial filtering method, is achieved by subtracting the mean signals of all electrodes from the selected electrode signals (Zheng et al., 2020d). Here, also choosing the Oz electrode, the weights ***w*** can be expressed as:

(12) $$\text{w}={\left[\frac{N_{e}-1}{N_{e}},-\frac{1}{N_{e}},\ldots ,-\frac{1}{N_{e}}\right]}^{\text{T}}.$$

#### Minimum Energy Combination

The MEC-based spatial filtering is proposed by Friman et al. (2007) to minimize the energy from nuisance signals. First, by removing any potential SSVEP activity from *N*_*e*_-electrodes signals ***Y*** by projecting them onto the orthogonal complement of the SSVEP model matrix ***X_f_*** in **Equation (3)**, the nuisance signals ${\tilde{\text{Y}}}_{f}\in {\mathbb{R}}^{N_{t}\times N_{e}}$ can be expressed as (Friman et al., 2007):

(13) $${\tilde{\text{Y}}}_{f}=\text{Y}-{\text{X}}_{f}{\left({\text{X}}_{f}^{\text{T}}{\text{X}}_{f}\right)}^{-1}{\text{X}}_{f}^{\text{T}}\text{Y}.$$

where ${\tilde{\text{Y}}}_{f}$ contains only nuisance signals and noise. In other words, ${\tilde{\text{Y}}}_{f}\approx \text{E}$.

Next is to find a weight vector ${\hat{\text{w}}}_{f}\in {\mathbb{R}}^{N_{e}\times 1}$ to minimize the energy of the combination of electrode signals ${\tilde{\text{Y}}}_{f}{\hat{\text{w}}}_{f}$:

(14) $${\hat{\text{w}}}_{f}=\underset{{\hat{w}}_{f}}{\text{argmin}}{∥{\tilde{\text{Y}}}_{f}{\hat{\text{w}}}_{f}∥}^{2}=\underset{{\hat{w}}_{f}}{\text{argmin}}{\hat{\text{w}}}_{f}^{\text{T}}{\tilde{\text{Y}}}_{f}^{\text{T}}{\tilde{\text{Y}}}_{f}{\hat{\text{w}}}_{f}.$$

The above minimization problem can be solved by decomposing the eigenvalues of the matrix ${\tilde{\text{Y}}}_{f}^{\text{T}}{\tilde{\text{Y}}}_{f}$, and the spatial filter weights ${\hat{\text{w}}}_{f}$ are defined by the eigenvector ***v***_1_ corresponding to the smallest eigenvalue λ_1_ (Friman et al., 2007; Yan et al., 2019):

(15) $${\hat{\text{w}}}_{f}=\frac{{\text{v}}_{1}}{\sqrt{\lambda_{1}}}$$

#### Maximum Contrast Combination

Maximum contrast combination is realized by maximizing the SSVEP energy and minimizing the nuisance noise energy simultaneously. Hence, MCC can be achieved as follows (Friman et al., 2007):

(16) $${\hat{\text{w}}}_{f}=\underset{{\hat{w}}_{f}}{\text{argmax}}\frac{{∥\text{Y}{\hat{\text{w}}}_{f}∥}^{2}}{{∥{\tilde{\text{Y}}}_{f}{\hat{\text{w}}}_{f}∥}^{2}}=\underset{{\hat{w}}_{f}}{\text{argmax}}\frac{{\hat{\text{w}}}_{f}^{\text{T}}{\text{Y}}^{\text{T}}\text{Y}{\hat{\text{w}}}_{f}}{{\hat{\text{w}}}_{f}^{\text{T}}{\tilde{\text{Y}}}_{f}^{\text{T}}{\tilde{\text{Y}}}_{f}{\hat{\text{w}}}_{f}}.$$

The above maxima can be found by a generalized eigen-decomposition of the matrices ***Y***^T^***Y*** and ${\tilde{\text{Y}}}_{f}^{\text{T}}{\tilde{\text{Y}}}_{f}$, and the spatial filter weights ${\hat{\text{w}}}_{f}$ are defined as the eigenvector corresponding to the largest eigenvalue (Zerafa et al., 2018).

#### Canonical Correlation Analysis

Canonical correlation analysis, a statistical way to measure the underlying correlation between two sets of multidimensional variables, was first used in SSVEP analysis by Lin et al. (2007). Till now, CCA has become the most widely used method in SSVEPs as a result of its effectiveness, robustness, and simple implementation (Bin et al., 2009; Zheng et al., 2020b; Li et al., 2021). Here, CCA finds the weights ***w_y_*** ∈ *ℝ*^*N*_*e*_×1^ and ***w_xf_*** ∈ *ℝ*^2*N*_*h*_×1^ to maximize the linear combinations between ***y*** = ***Y_wy_*** ∈ *ℝ*^*N*_*t*_×1^ and ***x*** = ***X_f_ w_xf_*** ∈ *ℝ*^*N*_*t*_×1^ representing the multichannel SSVEP signals and the SSVEP reference signals. Hence, the weight vectors ***w_y_*** and ***w_xf_*** can be obtained as follows:

(17) $$\begin{array}{c} {\text{w}}_{y},{\text{w}}_{xf}=\underset{W_{y},W_{xf}}{\text{argmax}}\rho \left(y,x\right)=\frac{\text{E}\left[{\text{y}}^{\text{T}}\text{x}\right]}{\sqrt{\text{E}\left[{\text{y}}^{\text{T}}\text{y}\right]\text{E}\left[{\text{x}}^{\text{T}}\text{x}\right]}}\\ \;=\frac{\text{E}\left[{\text{w}}_{y}^{\text{T}}{\text{Y}}^{\text{T}}{\text{X}}_{f}{\text{w}}_{xf}\right]}{\sqrt{\text{E}\left[{\text{w}}_{y}^{\text{T}}{\text{Y}}^{\text{T}}{\text{Yw}}_{y}\right]\text{E}\left[{\text{w}}_{\mathrm{xf}}^{\text{T}}{\text{X}}_{f}^{\text{T}}{\text{X}}_{f}{\text{w}}_{\mathrm{xf}}\right]}}. \end{array}$$

The maximum of ρ is the maximum canonical correlation. The spatial filter weights ***w_y_*** is defined as the eigenvector corresponding to the largest eigenvalue after transforming the above optimization problem into the eigenvalue decomposition problem (Yan et al., 2019).

#### Multivariate Synchronization Index

Multivariate synchronization index, introduced by Zhang et al. (2014a), is another multichannel detection method for SSVEPs. Assuming that the reference signal ***X_f_*** is synchronized to the SSVEP signals ***Y***, MSI is used for estimating the synchronization between ***Y*** and ***X_f_***. First, the matrices of***Y*** and ***X_f_*** are normalized to have a zero mean and unitary variance. Then, a correlation matrix ***C*** is estimated as (Zhang et al., 2014a):

(18) $$\text{C}=\left[\begin{array}{cc} {\text{C}}_{\mathrm{YY}}\; & {\text{C}}_{{\mathrm{YX}}_{f}}\\ {\text{C}}_{X_{f}Y}\; & {\text{C}}_{X_{f}X_{f}} \end{array}\right]$$

where

(19) $${\text{C}}_{\mathrm{YY}}=\frac{1}{N_{t}}{\text{YY}}^{\text{T}},{\text{C}}_{X_{f}X_{f}}=\frac{1}{N_{t}}{\text{X}}_{f}{\text{X}}_{f}^{\text{T}},{\text{C}}_{{\mathrm{YX}}_{f}}={\text{C}}_{X_{f}Y}=\frac{1}{N_{t}}{\text{YX}}_{f}^{\text{T}}.$$

To weaken the effect from the autocorrelation on the synchronization measure, the following linear transformation is adopted:

(20) $$\text{U}=\left[\begin{array}{cc} {\text{C}}_{\text{YY}}^{\text{-}1/2} & 0\\ 0 & {\text{C}}_{{\text{X}}_{\text{f}}{\text{X}}_{\text{f}}}^{\text{-}1/2} \end{array}\right]$$

The transformed correlation matrix ***C***′ is as follows after canceling out the autocorrelation:

(21) $${\text{C}}^{{}^{\prime }}={\text{UCU}}^{\text{T}}$$

Here, rather than the previous studies using the synchronization index S-estimator in MSI-based frequency recognition in SSVEPs (Zhang et al., 2014a; Zerafa et al., 2018), the spatial filter weights ***w*** is directly obtained by the eigenvector corresponding to the largest eigenvalue of the matrix ***C***′.

#### Partial Least Squares

Partial least squares is a commonly used multiple linear regression method to compute the linear regression between multidimensional predicted variables and multidimensional observable variables (Trejo et al., 2006; Wang et al., 2014a). Wang et al. (2014a) and Ge et al. (2017) proposed a double PLS-based recognition method in SSVEPs, where the first step is to use PLS as a spatial filter to enhance the SNR. Here, we mainly focused on the first step.

In PLS, the SSVEP signals ***Y*** and the reference signal ***X_f_*** are first decomposed into bilinear terms by an iterative procedure to extract the latent variables with maximal correlation (Rosipal and Krämer, 2006):

(22) $$\text{Y}={\text{TP}}^{\text{T}}+\text{E}$$

(23) $${\text{X}}_{f}={\text{UQ}}^{\text{T}}+\text{F}$$

where matrices $\text{T}={\left\{t_{i}\right\}}_{i=1}^{D}$ and $\text{U}={\left\{u_{i}\right\}}_{i=1}^{D}$ are the extracted *D* latent vectors (i.e., score vectors), ***P*** and ***Q*** are loading matrices, and ***E*** and***F*** are residual matrices. Since ***Y*** can be regarded as a linear mixture of ***X_f_*** and noise [see **Equation (4)**], ***X****_*f*_* can be decomposed by ***Y***:

(24) $${\text{X}}_{f}={\text{YW}}_{f}+{\text{F}}_{f}$$

where ***F***_*f*_ is the residual matrix. ***W***_*f*_ is the matrix of linear regression coefficients, which can be defined as (Rosipal and Krämer, 2006):

(25) $${\text{W}}_{f}={\text{Y}}^{\text{T}}\text{U}{\left({\text{T}}^{\text{T}}{\text{YY}}^{\text{T}}\text{U}\right)}^{-1}{\text{T}}^{\text{T}}{\text{X}}_{f}$$

The spatially filtered SSVEP signals ***S*** can be obtained by removing the residual matrix ***F***_*f*_:

(26) $$\text{S}={\text{YW}}_{f}$$

Here, the spatial filter weights ***w*** is obtained by the eigenvector corresponding to the largest eigenvalue of the matrix ***W***_*f*_.

## Experiment

### Participants

Eleven healthy volunteers (four female, ages 22–27 years) were recruited from Xi’an Jiaotong University. The subjective visual acuity was evaluated by Freiburg Visual Acuity and Contrast Test (FrACT) monocularly (Bach, 1996). The experimental protocol was approved by the Human Ethics Committee of Xi’an Jiaotong University, conforming to the Declaration of Helsinki. All subjects also submitted the written consent after informed of the contents of the experiment.

### Experimental Equipment

**FIGURE 1:**
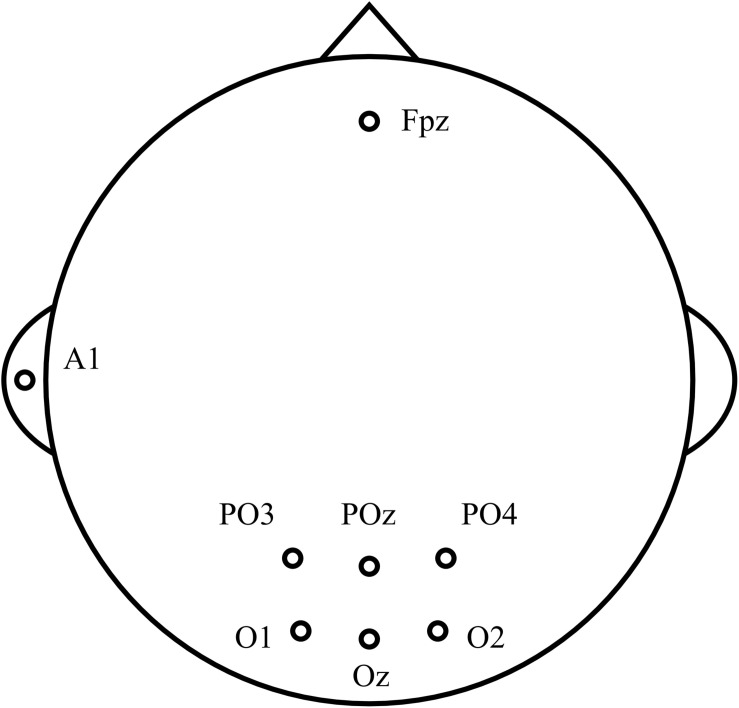
Location of scalp electrodes.

Electroencephalography was recorded by an EEG system (g.USBamp and g.GAMMAbox, g.tec, Schiedlberg, Austria) with a sampling frequency of 1,200 Hz. According to the previous studies (Hemptinne et al., 2018; Zheng et al., 2020a), six occipital electrodes (O1, Oz, O2, PO3, POz, and PO4) were used to acquire EEG signals, as shown in Figure 1. The ground electrode was placed on the forehead (Fpz), and the reference electrode was placed on the left earlobe (A1). Besides, a notch filter from 48 to 52 Hz was applied to eliminate the power line interference. A 24.5-in LCD monitor (PG258Q, ASUS, Taipei, China) with a resolution of 1,920 × 1,080 pixels, and a refresh rate of 240 Hz was used to present visual stimuli.

### Visual Stimuli

In this study, the vertical sinusoidal gratings with a reversal frequency of 7.5 Hz were used as the visual stimuli with the Michelson contrast of 50% and the mean background luminance of 80 cd/m^2^ (Kurtenbach et al., 2013; Zheng et al., 2020c). The visual angle of the stimulus pattern with a side length of 720 pixels was set as four degrees by adjusting the distance between the display and subjects. Six spatial frequencies in logarithmically equidistant steps of 3.0, 4.8, 7.5, 12.0, 19.0, and 30.0 cycles per degree (cpd) corresponding to the optotypes of 1.0, 0.8, 0.6, 0.4, 0.2, and 0.0 logMAR were presented to subjects in each run (Zheng et al., 2019). Each run contained six blocks corresponding to six spatial frequency steps. Each block contained five trials, and each trial lasted 5 s with a 2-s interval between two trials. The right eye was tested first and then the left eye. Besides, four subjects accomplished two eyes’ experiments, while the others only accomplished the right eye’s experiment. The visual stimuli were developed by MATLAB (MathWorks, Natick, MA, United States) using the Psychophysics Toolbox (Brainard, 1997).

### Signal Processing

#### Data Preprocessing

Following the start and end times of each trial, the SSVEP data segments were extracted. Then, a band-pass filter from 3 to 40 Hz was imposed to exclude the high-frequency interferences and low-frequency drifts. The five data segments of the same spatial frequency corresponding to five trials in one block were averaged to a 5-s data epoch for further data processing.

#### Spatial Filtering and Feature Extraction

The above 10 spatial filtering methods were used to linearly combine the 5-s six-electrode data epoch into 5-s single-channel signals, respectively. Since there was only one stimulus frequency, i.e., 7.5 Hz, in stimulus presentation, the SSVEP reference signals model ***X_f_*** ∈ *ℝ*^*N*_*t*_×2*N*_*h*_^ in this study was defined as:

(27) $${\text{X}}_{f}={\left(\begin{array}{c} \text{sin}\left(2\pi f\frac{m}{F_{s}}\right)\\ \text{cos}\left(2\pi f\frac{m}{F_{s}}\right) \end{array}\right)}^{\text{T}},m=1,\ldots ,N_{t}$$

where *f* was set as 7.5 Hz, and the number of harmonic frequencies *N*_*h*_ was set as 1. The number of sampling points, *N*_*t*_, was 6,000 in a 5-s data segment with a sampling frequency of 1,200 Hz.

Then, the SSVEP feature was extracted by the Fourier transform to obtain the frequency-domain spectrum, and the amplitude at the fundamental reversal frequency of 7.5 Hz was considered as the SSVEP amplitude.

#### Signal-to-Noise Ratio

The noise was defined by the mean value of the 20 adjacent amplitudes of either side of the fundamental frequency of 7.5 Hz on the frequency-domain spectrum (Bach and Meigen, 1999; Zheng et al., 2020b). Hence, the SNR can be determined by the ratio of SSVEP amplitude at 7.5 Hz to noise:

(28) $$\begin{array}{c} SNR=\frac{\mathrm{SSVEP}\mathrm{amplitude}}{\text{noise}}\\ \;=\frac{a\left(f\right)}{\frac{1}{10}*\sum_{k=1}^{k=10}a\left(fk*△f\right)a\left(f-k*△f\right)} \end{array}$$

where *a(f)* denotes the amplitude on the frequency-domain spectrum at frequency *f*, and frequency resolution Δ*f* is 0.1 Hz.

### Visual Acuity Determination Criterion

**FIGURE 2:**
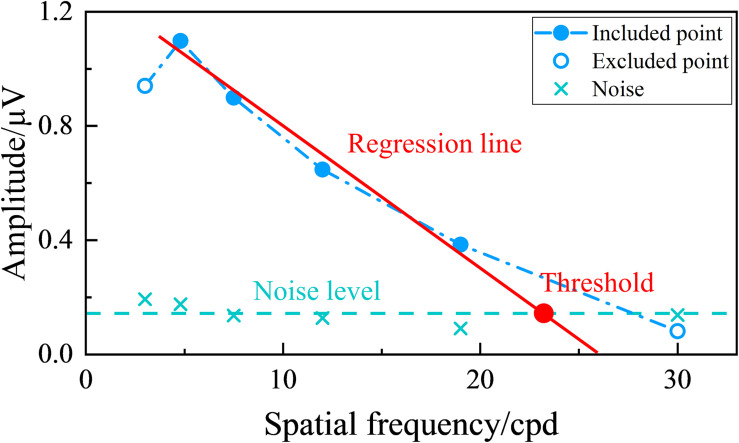
Example of tuning curve for steady-state visual evoked potential (SSVEP) visual acuity estimation criterion. The green “ × ” represents the noise corresponding to each spatial frequency step, and the green dashed line represents the noise level baseline defined by the mean of the noise of the six spatial frequency steps. The data points included in the linear regression have an signal-to-noise ratio (SNR) higher than the preset SNR level, while the excluded points do not. The red solid line represents the regression line between the SSVEP amplitude and spatial frequency extrapolating from the last significant SSVEP peak to the last data point with an SNR higher than the preset SNR level. The red point is the intersection of the regression line and the noise level baseline, with its corresponding spatial frequency value defined as the visual acuity threshold.

**FIGURE 3:**
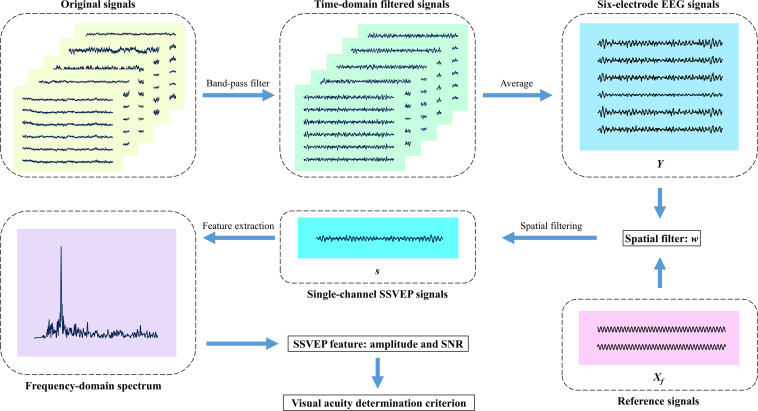
Diagram of signal processing in this study. First, the original signals for five trials in one block are filtered by a band-pass filter from 3 to 40 Hz, and subsequently, the signal segments corresponding to five trials are averaged to a 5-s data epoch for each electrode. Then, each spatial filtering method linearly combines the six-electrode signals into one single-channel signals, respectively. Next, the Fourier transform extracts the steady-state visual evoked potential (SSVEP) amplitude and signal-to-noise ratio (SNR). Finally, the visual acuity determination criterion is carried out after six blocks in one run complete. Besides, the model-based spatial filter is obtained by mathematical transformation of six-electrode EEG signals *Y* and reference signals *X*_*f*_.

Figure 2 shows an example of the tuning curve for the SSVEP visual acuity estimation criterion used in this study. SSVEP amplitude can be plotted versus spatial frequency, and then a regression line can be extrapolated from the last significant SSVEP peak to a noise level baseline (Zheng et al., 2020b). The range for the regression line was between the last significant SSVEP peak and the last data point with an SNR higher than the preset SNR level, and the noise level baseline for each visual stimulus was defined as the mean of the noise of the six spatial frequency steps (Hamilton et al., 2021b). Then, the SSVEP visual acuity was defined as the spatial frequency corresponding to the intersection point between the regression line and the noise level baseline (Zheng et al., 2020b; Hamilton et al., 2021a). Besides, the whole diagram of signal processing in this study is shown in Figure 3.

### Statistical Analysis

Bland–Altman was used to describe the agreement and difference between the psychophysical FrACT and objective SSVEP visual acuity for each spatial filtering method. Besides, one-way repeated-measures ANOVA was also employed to evaluate the difference among the FrACT and SSVEP visual acuity results for each spatial filtering method, and the *post-hoc* analysis with Bonferroni correction for multiple comparisons was subsequently employed.

## Results

### Comparison of the SSVEP Signal Characteristics

**FIGURE 4:**
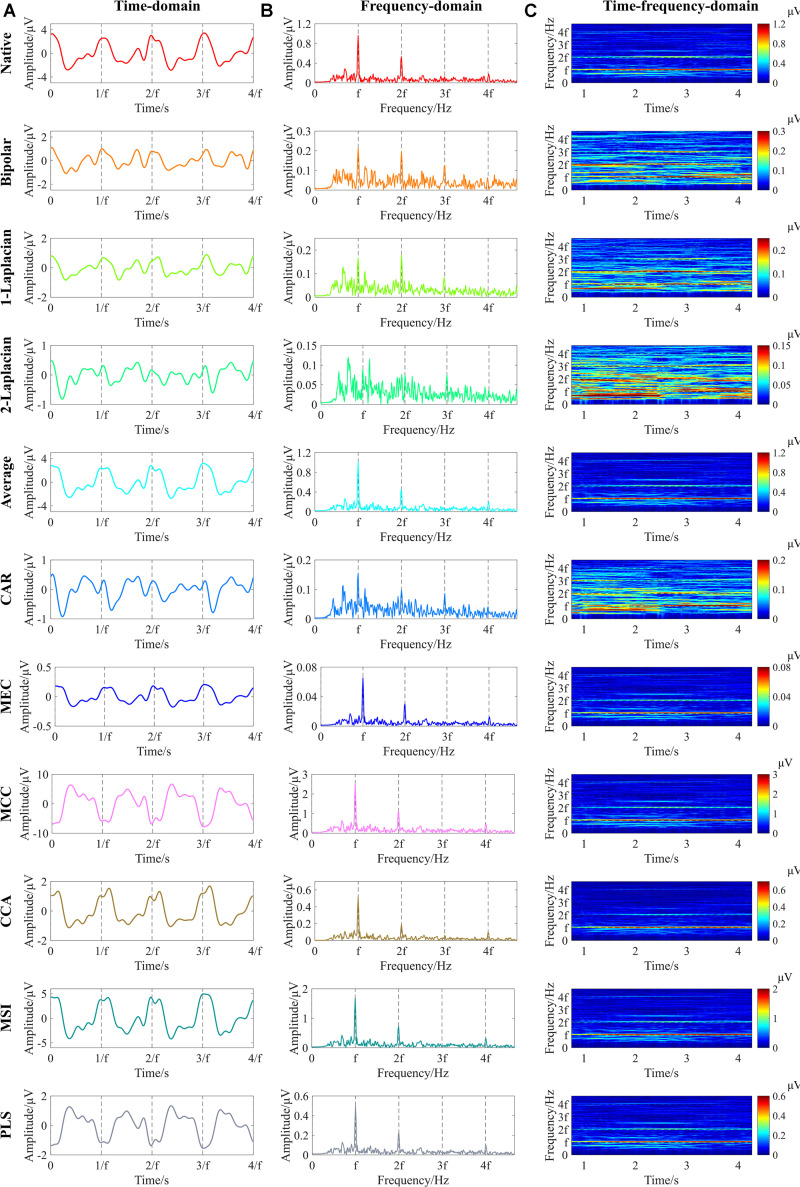
Example of the time-domain, frequency-domain, and time–frequency-domain analyses of steady-state visual evoked potentials (SSVEPs) at 3.0 cpd after various spatial filtering methods (right eye of subject S9, Freiburg Visual Acuity and Contrast Test (FrACT) acuity = 0.00 logMAR). **(A)** Time-domain analysis. The vertical dashed lines correspond to four periods of the reversal process. **(B)** Frequency-domain analysis. **(C)** Time–frequency-domain analysis. The vertical dashed lines in Panel **(B)** and the horizontal dashed lines in Panel **(C)** correspond to the reversal frequency of 7.5 Hz and the second, third, and fourth harmonic frequencies of 15, 22.5, and 30 Hz, respectively. “f” in all subfigures represents the reversal frequency of 7.5 Hz.

Figure 4 shows an example of the time-domain, frequency-domain, and time–frequency-domain analyses of SSVEPs after each spatial filtering method. First, the 5-s single-channel SSVEP signals corresponding to each spatial filtering method were obtained according to the abovementioned signal processing flow in Figure 3. Then, the time-domain waveforms were obtained by averaging the 0.53-s nonoverlapping data segments subdivided by the 5-s single-channel SSVEP signals, with each segment containing four periods of the reversal process (Zheng et al., 2020a). The frequency-domain spectrums were obtained by the Fourier transform of the 5-s single-channel SSVEP signals. As for the time–frequency-domain analysis, the 2.0-s window length with 0.1-s sliding length over the 5-s single-channel signals was used to obtain the time–frequency-domain characteristics (Zheng et al., 2020a).

The time-domain waveforms in Figure 4A show that an obvious main periodicity was the fundamental reversal frequency of 7.5 Hz for all spatial filtering methods except for the two-dimensional Laplacian combination, while some other periodic components also existed in some waveforms, such as the native, bipolar, and one-dimensional Laplacian combination. Both the frequency-domain waveforms in Figure 4B and the time–frequency-domain analyses in Figure 4C show clear significant peaks at the fundamental reversal frequency of 7.5 Hz and the second harmonic frequency of 15 Hz for all spatial filtering methods except for the two-dimensional Laplacian combination, indicating that all these spatial filtering methods except for the two-dimensional Laplacian combination can obtain obvious signal characteristics by combining the multielectrode signals into single-channel signals.

### Comparison of Spatial Filtering Effect

**FIGURE 5:**
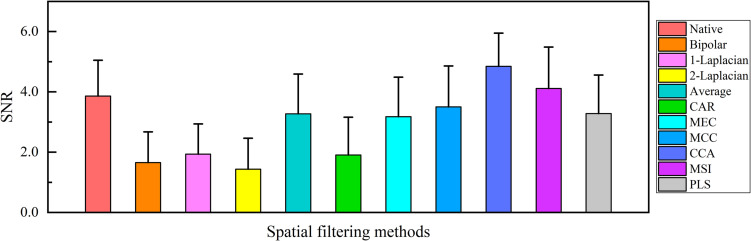
Comparison of the mean values and SD of the signal-to-noise ratio (SNR) of the single-channel steady-state visual evoked potential (SSVEP) signals corresponding to various spatial filtering methods at 3.0 cpd over all subjects.

The main purpose of spatial filtering is to strengthen the SSVEP components and suppress the non-SSVEP components in EEG signals (Wong et al., 2020) and thus to enhance the SNR (Friman et al., 2007). Hence, the spatial filtering effect was evaluated by comparing the SNR values of the single-channel SSVEP signals corresponding to various spatial filtering methods. Since the visual stimuli at the spatial frequency of 3.0 cpd were the clearest to all subjects, the comparison of the SNR values corresponding to various spatial filtering methods at 3.0 cpd over all subjects was obtained, as shown in Figure 5. Figure 5 shows that the SNR values of CCA (4.849 ± 1.101) and MSI (4.115 ± 1.372) were higher than that of the native combination (3.861 ± 1.188), with other spatial filtering methods had lower or close SNR values to that of the native combination. Since the native combination actually utilized only single-electrode signals from Oz and was widely used in SSVEP visual acuity assessment, here, the spatial filtering methods of CCA and MSI were compared to the native combination in the further visual acuity evaluation by SSVEPs.

### SSVEP Visual Acuity Threshold Determination Criterion

**FIGURE 6:**
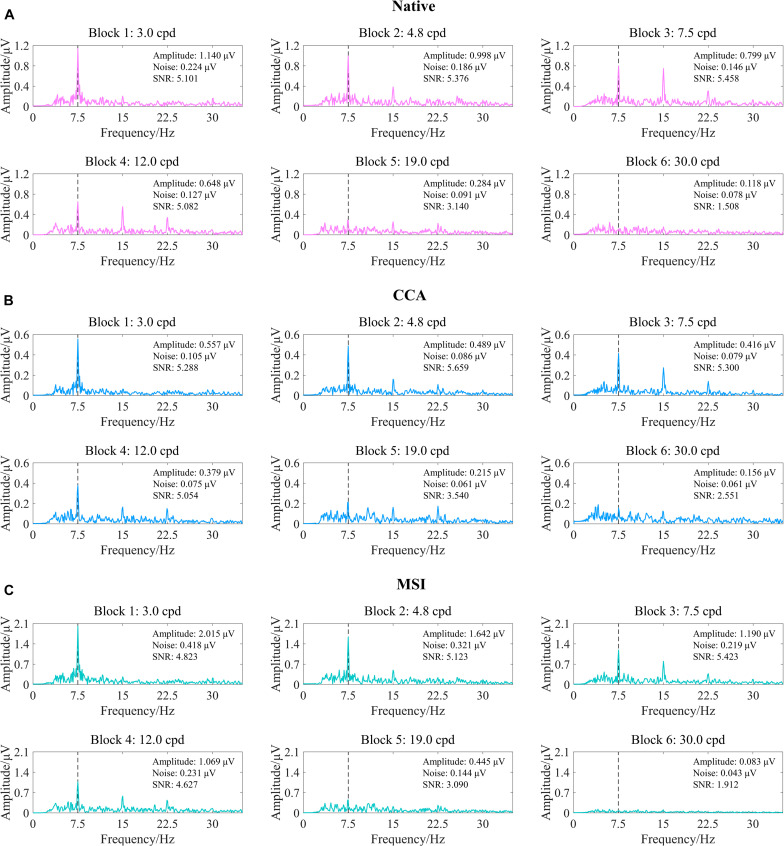
Examples of the steady-state visual evoked potential (SSVEP) response to six spatial frequency steps after three spatial filtering methods of native combination, canonical correlation analysis (CCA), and multivariate synchronization index (MSI) (right eye of subject S2, FrACT acuity = −0.06 logMAR). **(A)** Native combination. **(B)** CCA. **(C)** MSI. The vertical dashed lines correspond to the reversal frequency of 7.5 Hz.

SSVEP visual acuity was defined by the intersection point between the noise level baseline and the regression line extrapolating from the last significant SSVEP peak to the last data point with an SNR higher than the preset SNR level. For the native combination, previous studies have given the recommended value of SNR level, i.e., 1.0 (Yadav et al., 2009; Zheng et al., 2020b). However, as shown in Figure 6, CCA and MSI often obtained the higher SNR of SSVEPs than the native combination, especially in high spatial frequencies close to the visual acuity threshold. Hence, for the spatial filtering methods of CCA and MSI, the SNR level of 1.0 may not be applicable since both CCA and MSI enhanced the SNR of SSVEPs.

**FIGURE 7:**
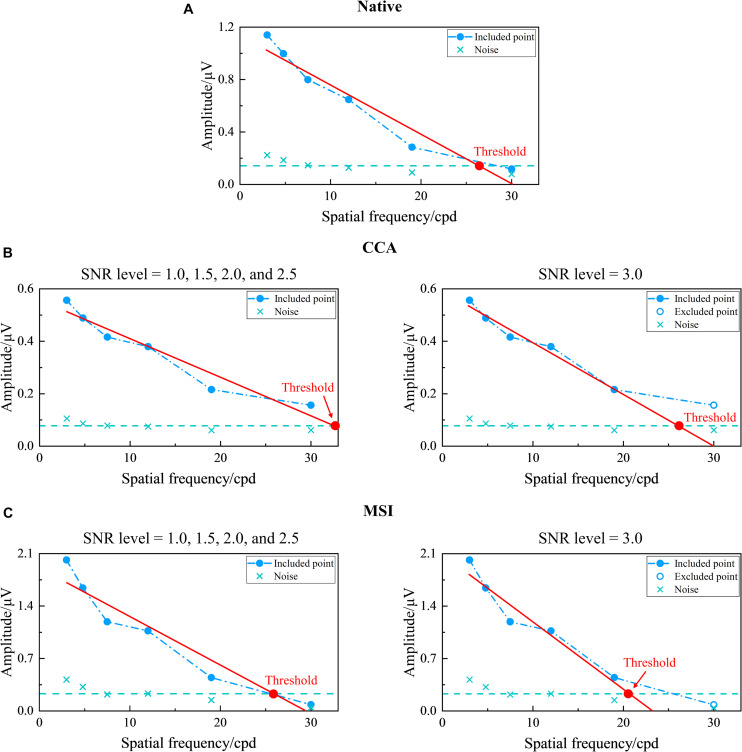
Examples of the tuning curves for steady-state visual evoked potential (SSVEP) visual acuity estimation criterion corresponding to the native combination, canonical correlation analysis (CCA), and multivariate synchronization index (MSI) with various signal-to-noise ratio (SNR) levels of 1.0, 1.5, 2.0, 2.5, and 3.0, respectively (right eye of subject S2, FrACT acuity = −0.06 logMAR). **(A)** Native combination with the SNR level of 1.0. **(B)** CCA with the SNR levels of 1.0, 1.5, 2.0, and 2.5 for the left subpanel and 3.0 for the right subpanel. **(C)** MSI with the SNR levels of 1.0, 1.5, 2.0, and 2.5 for the left subpanel and 3.0 for the right subpanel. The representations of the symbols and lines are the same as in Figure 2.

Here, first, the five SNR levels, i.e., 1.0, 1.5, 2.0, 2.5, and 3.0 (Zheng et al., 2019), were preselected for CCA and MSI. Then, as shown in Figure 7, corresponding to Figure 6, the tuning curves of the SSVEP visual acuity estimation criterion for the native combination, CCA, and MSI with various SNR levels of 1.0, 1.5, 2.0, 2.5, and 3.0, respectively, can be obtained. Next, the range for the linear regression of the native combination in Figure 7A was from the first data point with the amplitude peak of 1.140 μV to the last data point with an SNR of 1.508 higher than the SNR level of 1.0, and the SSVEP visual acuity for the native combination was determined as the spatial frequency of the intersection point of the regression line and the noise level baseline, i.e., 26.554 cpd. Similar to this, as shown in Figures 7B,C, the SSVEP visual acuities for CCA and MSI with various SNR levels were 32.470 cpd for CCA with the SNR levels of 1.0, 1.5, 2.0, and 2.5; 26.097 cpd for CCA with the SNR level of 3.0; 25.237 cpd for MSI with the SNR levels of 1.0, 1.5, 2.0, and 2.5; and 20.892 cpd for MSI with the SNR level of 3.0.

The unit of logMAR was used in the final visual acuity expression for its uniformity in spatial frequency (Bach, 2007). Finally, after SSVEP visual acuities for CCA and MSI at various SNR levels over all subjects were obtained, the Bland–Altman analysis was used to analyze the difference and agreement between subjective FrACT visual acuity and objective SSVEP visual acuity for CCA and MSI at each SNR level, as shown in Table 1. Hence, the SNR level of 2.0 was chosen for CCA with a low 95% limit of agreement (i.e., 0.202 logMAR) and a low difference (i.e., 0.039 logMAR). Similarly, the SNR level of 1.5 was chosen for MSI with a low 95% limit of agreement (i.e., 0.208 logMAR) and a low difference (i.e., −0.080 logMAR).

**TABLE 1:** Results of Bland–Altman analysis between subjective Freiburg Visual Acuity and Contrast Test (FrACT) visual acuity and objective steady-state visual evoked potential (SSVEP) visual acuity for the native combination at signal-to-noise ratio (SNR) level of 1.0, and canonical correlation analysis (CCA) and multivariate synchronization index (MSI) at each SNR level of 1.0, 1.5, 2.0, 2.5, and 3.0, respectively.

	SNR level	Difference/logMAR	LoA/logMAR
Native	1.0	−0.095	0.253
CCA	1.0	0.057	0.204
	1.5	0.050	0.215
	2.0	0.039	0.202
	2.5	−0.011	0.230
	3.0	−0.065	0.348
MSI	1.0	−0.902	0.254
	1.5	−0.080	0.208
	2.0	−0.158	0.290
	2.5	−0.269	0.304
	3.0	−0.298	0.256

*LoA, 95% limit of agreement.*

### Comparison of Visual Acuity Results

**FIGURE 8:**
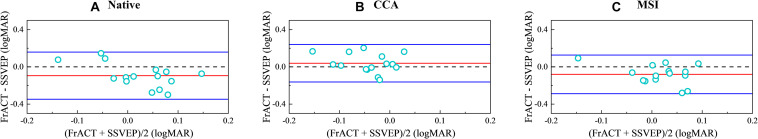
Bland–Altman analysis between psychophysical Freiburg Visual Acuity and Contrast Test (FrACT) visual acuity and objective steady-state visual evoked potential (SSVEP) visual acuity over all subjects for the native combination, canonical correlation analysis (CCA), and multivariate synchronization index (MSI), respectively. **(A)** Native combination. **(B)** CCA. **(C)** MSI. In each panel, the red solid line represents the average value of the difference. The blue solid lines represent the 95% limit of agreement. The dashed line represents the difference of zero.

Figure 8 shows the Bland–Altman analysis between subjective FrACT visual acuity and final objective SSVEP visual acuity over all subjects for the native combination, CCA, and MSI, respectively. The 95% limits of agreement for the native combination, CCA, and MSI were 0.253 logMAR, 0.202 logMAR, and 0.208 logMAR, respectively, indicating that SSVEP visual acuity of the spatial filtering methods of CCA and MSI had better accuracy than the native combination.

**FIGURE 9:**
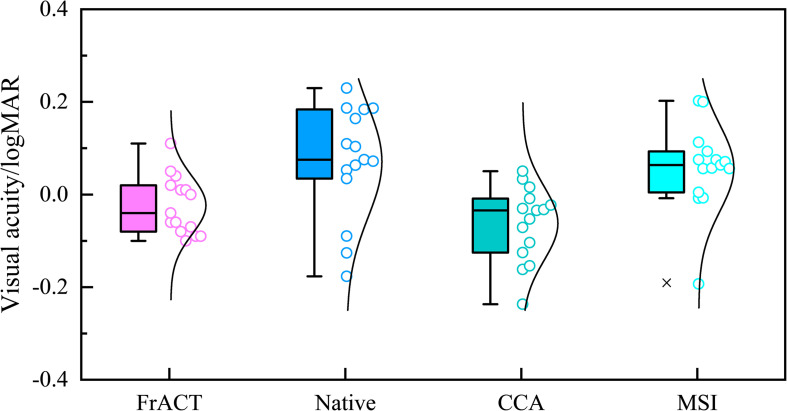
Comparison of the visual acuity assessed by Freiburg Visual Acuity and Contrast Test (FrACT) and steady-state visual evoked potentials (SSVEPs) from three spatial filtering methods of the native combination, canonical correlation analysis (CCA), and multivariate synchronization index (MSI) over all subjects.

Figure 9 shows the comparison in visual acuity estimated by four methods, i.e., FrACT and SSVEPs for three spatial filtering methods of the native combination, CCA, and MSI, over all subjects. One-way repeated-measures ANOVA found a significant difference in visual acuity among these four methods [*F*_(3, 45)_ = 10.277, *p* < 0.001]. Then, Bonferroni *post-hoc* analysis showed no difference between psychophysical FrACT visual acuity and each SSVEP visual acuity for the native combination, CCA, and MSI (*p* > 0.05), as shown in Table 2, demonstrating that the SSVEP visual acuity obtained by these three spatial filtering methods all had a good agreement and a similar performance with subjective FrACT visual acuity. Besides, a significantly higher SSVEP visual acuity was found in CCA than the native combination (*p* = 0.005) and MSI (*p* < 0.001), indicating CCA had a better performance in combining the multielectrode signals in SSVEPs, especially when the spatial frequency near the psychophysical threshold, causing the higher SNR, i.e., higher SSVEP amplitude and lower noise, as shown in Figure 5.

**TABLE 2:** Bonferroni *post hoc* analysis of visual acuity among Freiburg Visual Acuity and Contrast Test (FrACT) and steady-state visual evoked potentials (SSVEPs) from three spatial filtering methods of the native combination, canonical correlation analysis (CCA), and multivariate synchronization index (MSI).

Method	Native	CCA	MSI
FrACT	*p* = 0.061	*p* = 0.522	*p* = 0.096
Native	–	*p* = 0.005**	*p* = 1.000
CCA	–	–	*p* < 0.001***

***p < 0.001; **p < 0.01.

In summary, compared to FrACT visual acuity, SSVEP visual acuity for the native combination, CCA, and MSI all had a good agreement with it, demonstrating that these three spatial filtering methods all had a good performance in SSVEP visual acuity assessment. Besides, CCA-based SSVEP visual acuity had a better performance than MSI and the native combination, with a difference and a limit of agreement of 0.039 logMAR and 0.202 logMAR, respectively, lower than −0.080 logMAR and 0.208 logMAR for MSI and −0.095 logMAR and 0.253 logMAR for the native combination, as shown in Table 1. Hence, this study recommended CCA as the spatial filtering method for multielectrode signals combination in the SSVEP visual acuity assessment.

## Discussion

In this study, to enhance the performance of visual acuity by SSVEPs, 10 commonly used spatial filtering methods, i.e., native combination, bipolar combination, Laplacian combination, average combination, CAR, MEC, MCC, CCA, MSI, and PLS, were compared to combine multielectrode SSVEP signals into single-channel SSVEP signals for the vertical sinusoidal gratings, finding that the Fourier analysis of SSVEP signals after these 10 spatial filtering methods all had a significant peak at the fundamental reversal frequency, where CCA- and MSI-based SSVEP signals had a higher SNR than the traditional single-electrode from Oz, i.e., the native combination. Then, CCA and MSI were used in the further SSVEP visual acuity evaluation. Compared to the SNR level of 1.0 for the native combination, according to the Bland–Altman analysis, the SNR levels of 2.0 and 1.5 were chosen for CCA and MSI, respectively, to determine the regression range for visual acuity determination criterion. After the calculation of SSVEP visual acuity over all subjects, SSVEP visual acuity for the native combination, CCA, and MSI all had a good agreement with subjective FrACT visual acuity, with CCA-based SSVEP visual acuity realizing the best performance, recommending CCA as the spatial filtering method for multielectrode signals combination in SSVEP visual acuity assessment.

The CCA-based SSVEP visual acuity achieved a difference of 0.039 logMAR and a limit of agreement of 0.202 logMAR from FrACT visual acuity, and that for MSI-based SSVEP visual acuity were −0.080 logMAR and 0.208 logMAR, which was all lower than them of SSVEP visual acuity for the native combination with a difference and a limit of agreement of −0.095 logMAR and 0.253 logMAR. Since the spatial filtering methods can enhance the SNR of SSVEPs and suppress the non-SSVEP noise (Nakanishi et al., 2018b), this result illustrated that the unrelated noise, e.g., EMG and EOG (Friman et al., 2007; Zhang et al., 2021), was one of the reasons for the difference between SSVEP and behavioral visual acuity (Hamilton et al., 2021b), and the other methods of enhancing the SNR, such as signal preprocessing (Kołodziej et al., 2016), e.g., time-domain filtering (Zheng et al., 2021) and blind source separation (BSS) (Ji et al., 2019), and SSVEP recognition algorithms (Zhang et al., 2021), e.g., wavelet transform (WT) (Rejer, 2017) and empirical mode decomposition (EMD) (Huang et al., 2013; Tello et al., 2014), may also have the property to improve the agreement between SSVEP and behavioral visual acuity.

The 10 commonly used spatial filtering methods in this study can be divided into two categories. One is the basic spatial filtering methods canceling the common noise of each electrode via averaging or subtracting (Friman et al., 2007), such as native combination, bipolar combination, Laplacian combination, average combination, and CAR, and the other is called model-based spatial filtering methods using the mathematical transformation between multielectrode SSVEP signals and the SSVEP reference signals l (Zerafa et al., 2018), such as MEC, MCC, CCA, MSI, and PLS. Figure 5 shows that the model-based spatial filtering methods generally had a better performance than the basic spatial filtering methods in vertical sinusoidal gratings except for the average combination (Friman et al., 2007), and the reason for this may be that the model-based spatial filtering methods can adjust the weight coefficients to each electrode adaptively for various SSVEP signals.

All the spatial filtering methods used in this study were the training-free methods (Wong et al., 2020), which did not require any training data, and a new user can use this brain–computer interface (BCI) system immediately (Zerafa et al., 2018). Because of the fast and accurate requirement and infrequent testing for visual acuity assessment (Zheng et al., 2021), the training-free methods were adequate here. The filter bank strategy in training-free methods, such as filter bank CCA (FBCCA) (Chen et al., 2015) and filter bank MSI (FBMSI) (Qin et al., 2021), may be also used to enhance the performance of SSVEP-based visual acuity assessment in future work. In contrast, the subject-specific training methods with the best performance (Zerafa et al., 2018), requiring training data from the specific user and needing the cost of long and tiring training sessions, such as individual template-based CCA (itCCA) (Bin et al., 2011), combined-CCA (Nakanishi et al., 2014; Wang et al., 2014b), multiway CCA (Zhang et al., 2011), multiset CCA (Zhang et al., 2014b), and task-related component analysis (TRCA) (Nakanishi et al., 2018a), may be more suitable for the situation where the subjects need long-term use of BCI system, such as the vision training with SSVEP biofeedback in amblyopia (Lapajne et al., 2020). Besides, the subject-independent training methods requiring training data from various subjects, providing a good trade-off between training effort and performance (Zerafa et al., 2018), such as transfer template CCA (ttCCA) (Yuan et al., 2015) and combined-tCCA (Waytowich et al., 2016), may be further applied in SSVEP visual acuity assessment.

As for the threshold determination criterion in this study, the extrapolation technique by extrapolating a regression line between significant SSVEP amplitudes and spatial frequencies to a noise level baseline was used. Compared to the threshold determination criterion of the finest spatial frequency evoking a significant SSVEP (Hamilton et al., 2021a), where the precision depends on the sampling density of spatial frequency when near the threshold (Hamilton et al., 2021b), this extrapolation technique is more practical (Zheng et al., 2020b). Compared to the other stimulus paradigms, such as concentric rings with oscillating expansion and contraction (Zheng et al., 2019), the visual stimulus paradigm of vertical sinusoidal gratings in this study can easily be realized, as recommended by the International Society for Clinical Electrophysiology of Vision (ISCEV) standard (Hamilton et al., 2021a).

Here, the basic spatial filtering methods used the fixed reference electrode, Oz, for all subjects, but this may not necessarily be the best choice for each subject (Yan et al., 2021), so an adaptive reference electrode selection method may be explored in future work to improve the performance. In the model-based spatial filtering methods, only the eigenvector corresponding to one extreme value was chosen as spatial filter weights, e.g., the spatial filter weights corresponding to the largest eigenvalue in CCA, and there may be also some more signal information at eigenvectors of the second largest eigenvalue or even the latter eigenvalues (Zhao et al., 2020). Hence, future work may propose more algorithm strategies to make full use of the information from the spatial filtering methods. Finally, some subjects with lower visual acuity rather than the normal visual acuity may be also required for further research.

## Conclusion

This study introduced the spatial filtering methods in SSVEP-based visual acuity assessment, finding that CCA-based SSVEP visual acuity had a better performance with an agreement of 0.202 logMAR and a difference of 0.039 logMAR, compared to the single electrode and other spatial filtering methods. The study proved that the performance of SSVEP-based visual acuity can be enhanced by spatial filtering methods and also recommended CCA as the spatial filtering method for multielectrode signals combination in the SSVEP visual acuity assessment.

## Data Availability Statement

The raw data supporting the conclusions of this article will be made available by the authors, without undue reservation.

## Ethics Statement

The studies involving human participants were reviewed and approved by Human Ethics Committee of Xi’an Jiaotong University. The patients/participants provided their written informed consent to participate in this study.

## Author Contributions

XZ contributed to the study design, data acquisition, analysis, interpretation, manuscript writing, and revision. GX contributed to the study design and the approval of the final version for publication. CH and PT contributed to the statistical analysis and manuscript drafting. KZ and RL contributed to the data analysis and interpretation. YJ and WY contributed to the manuscript writing and revision. CD provided the experimental equipment and approved the final version for publication. SZ conceptualized the study. All authors contributed to the article and approved the submitted version.

## Conflict of Interest

The authors declare that the research was conducted in the absence of any commercial or financial relationships that could be construed as a potential conflict of interest.

## Publisher’s Note

All claims expressed in this article are solely those of the authors and do not necessarily represent those of their affiliated organizations, or those of the publisher, the editors and the reviewers. Any product that may be evaluated in this article, or claim that may be made by its manufacturer, is not guaranteed or endorsed by the publisher.
